# Role of Neoadjuvant Chemotherapy in Ovarian Serous Cancer Followed by Debulking

**DOI:** 10.7759/cureus.28909

**Published:** 2022-09-07

**Authors:** Sonali Chauhan, Deepti Shrivastava, Rajasbala Dhande, Asawari Deo

**Affiliations:** 1 Department of Obstetrics and Gynaecology, Jawaharlal Nehru Medical College, Datta Meghe Institute of Medical Science (Deemed to be University), Wardha, IND; 2 Department of Radiology, Jawaharlal Nehru Medical College, Datta Meghe Institute of Medical Science (Deemed to be University), Wardha, IND

**Keywords:** laparotomy, neoadjuvant chemotherapy, ca-125 levels, abdominal pain, ovary, serous cystadenocarcinoma

## Abstract

Background: Ovarian cancers are one of the most common gynecological cancers and serous tumor is one of the most common histological form of extrauterine female genital tract tumors. While ovarian serous carcinoma is a well-studied human gynecologic malignancy, this high-grade tumor remains lethal.

Case description: A 50-year-old female with P2L2A2 (Para-2, Live-2, Abortion-2) presented with pain in the abdomen for six months. Investigations were done, which revealed bilateral large ovarian cystic lesion suggestive of ovarian malignancy. She underwent six cycles of chemotherapy followed by exploratory laparotomy.

Objective: We examined the precipitating factors, laboratory abnormalities including cancer antigen 125 (CA-125) levels, treatment strategies including neoadjuvant therapy and debulking surgery, and clinical recovery in ovarian malignancy.

Conclusion: Primary debulking surgery (PDS), although the preferred treatment for ovarian cancer, is accompanied by combination chemotherapy based on platinum. However, neoadjuvant chemotherapy (NACT) followed by interval debulking surgery (IDS) has gained a reputation as a legitimate therapeutic technique specifically for patients with stage IV unresectable bulky tumors or poor general condition. Treatment with NACT is now expected to become a routine treatment or a successful treatment choice for advanced epithelial ovarian cancer (EOC).

## Introduction

Twenty-five percent of serous tumors are responsible for serous ovarian cystadenocarcinomas [1]. They account for the highest proportion of malignant ovarian tumors, accounting for over 50-80% of all malignant ovarian epithelial tumors [2]. The prevalence peaks in the sixth to seventh decades of life [3-5]. The only routine biomarker used to assess the status of the disease and monitor the success of chemotherapy in patients with epithelial ovarian cancer is cancer antigen-125 (CA-125). The value of CA-125 is expected to correlate with neoadjuvant chemotherapy (NACT) response in the neoadjuvant situation, where the effects of surgery are absent [4-6]. We present a case of serous papillary cystadenocarcinoma of the ovary in a 50-year-old patient and its treatment by NACT and debulking surgery [7,8].

## Case presentation

A 50-year-old woman presented in the outpatient department with severe abdominal pain, especially in the left iliac region, for the past six months. The pain was dull aching in nature and continuous and used to be relieved with oral analgesics. The patient also complained of shortness of breath, which worsened in the supine position and was eased in the sitting posture. She also stated that she had lost her appetite and lost a large amount of weight. There was no history of vaginal bleeding. There was no previous medical or surgical history.

On physical examination, the patient looked cachexic with mild degree of pallor with stable vital parameters. Abdomen examination showed a mass of 20 weeks of gestation size with irregular surface, heterogeneous consistency, tender, margins well defined but the lower pole was usually not reached, with restricted mobility. Ascites was present and positive shifting dullness was noted. A vaginal examination showed nodules on the right posterior fornix, tenderness was present. Left fornix was free.

**Figure 1:**
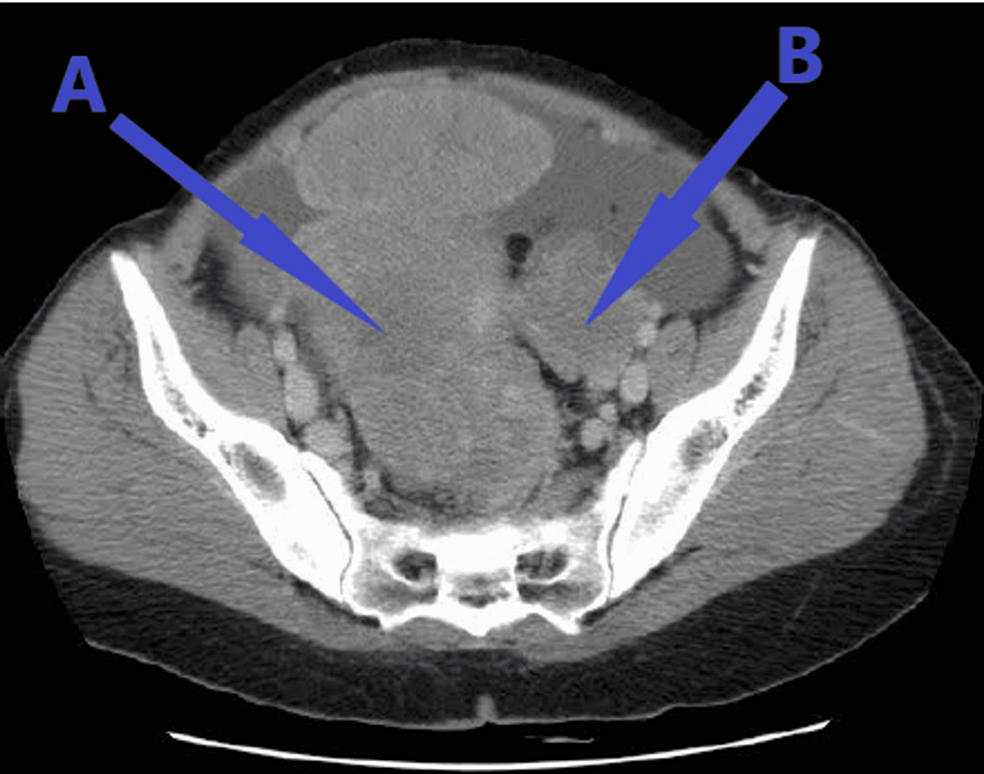
CECT of the abdomen showing extensive ascites with peritoneal carcinomatosis and bilateral heterogeneous enhancing ovarian mass. Arrows marked A and B show bilateral ovarian mass with gross ascites. CECT: contrast-enhanced computed tomography

The patient was advised for contrast-enhanced computed tomography (CECT) of the abdomen and pelvis, which revealed a large cystic lesion with peripheral enhancing solid areas in the pelvis in midline, probably arising from the left ovary, a possibility of malignant ovarian lesion (Figure 1). Another heterogeneously enhancing lesion was seen in the right adnexa. The right ovary was not seen separately from the lesion. There was a large ill-defined heterogeneously enhancing central mesenteric lesion with omental extension showing dense calcification, most likely metastatic deposit. Loculated ascites and left-sided moderate pleural effusion with few peripheral lung parenchymal nodules in the right middle and bilateral lower lobes were suggestive of stage 4A of the International Federation of Gynecology and Obstetrics (FIGO) system 2014.

**Figure 2:**
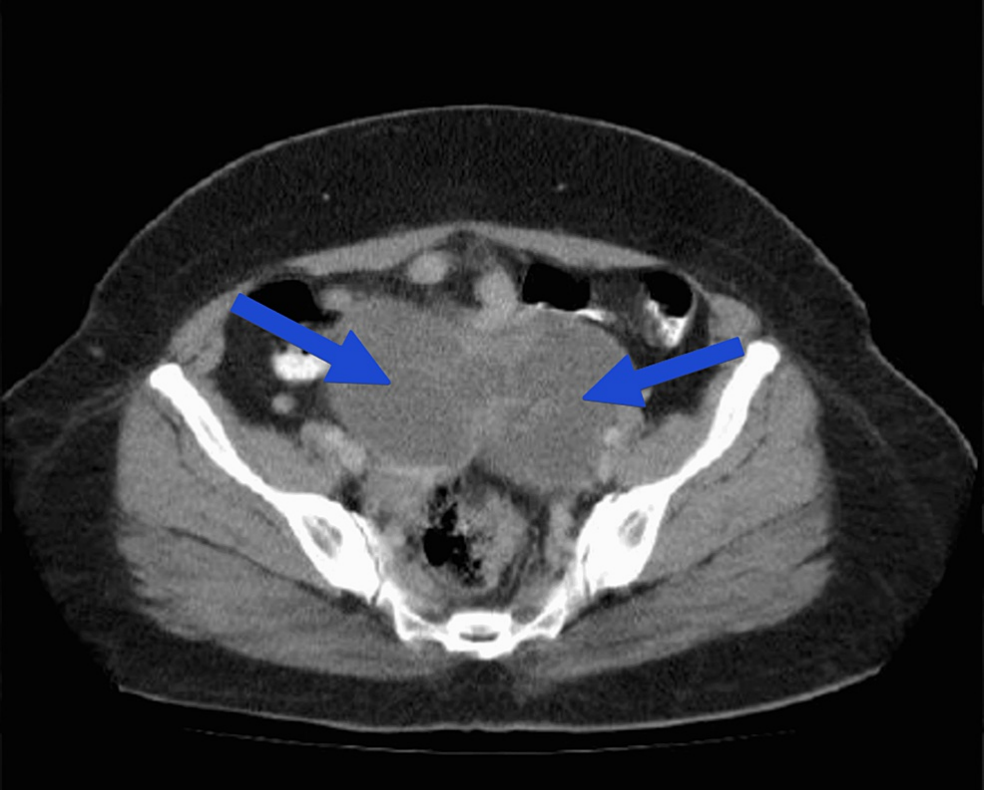
CECT abdomen showing bilateral ovarian masses. No ascites/omental caking detected. Arrows show bilateral ovarian mass.

CA-125 (cancer antigen 125) value was more than 1000 units/ml. NACT was given to the patient with paclitaxel 175 mg/m^2^ and carboplatin 675 mg/m^2^ (area under the curve (AUC)=5-6, calculated by Calvert’s formula). After two cycles of NACT with intervals of three weeks, CA-125 was 452 units/ml. The patient then underwent further four cycles of NACT. CA-125 was repeated, which showed normal values, and CECT was done, which revealed a complex cystic lesion in the pelvis and the infra-umbilical region at midline and para midline locations (Figure 2). A lesion of 2-3 cm thickness and peripherally enhancing solid mass of 8.6 x 5.8 cm in size was displacing the adjacent bowel loop. There was para midline location-significant regression in size of the mass. There was significant regression in ascites.

USG revealed a solid cystic lesion in the left adnexa measuring 5 x 4.2 x 3.6 cm; the solid component showed vascularity. Left ovary not seen separately. Findings were consistent with left ovarian complex cyst-neoplastic etiology. Omental thickening was seen in midline in the epigastric region.

**Figure 3:**
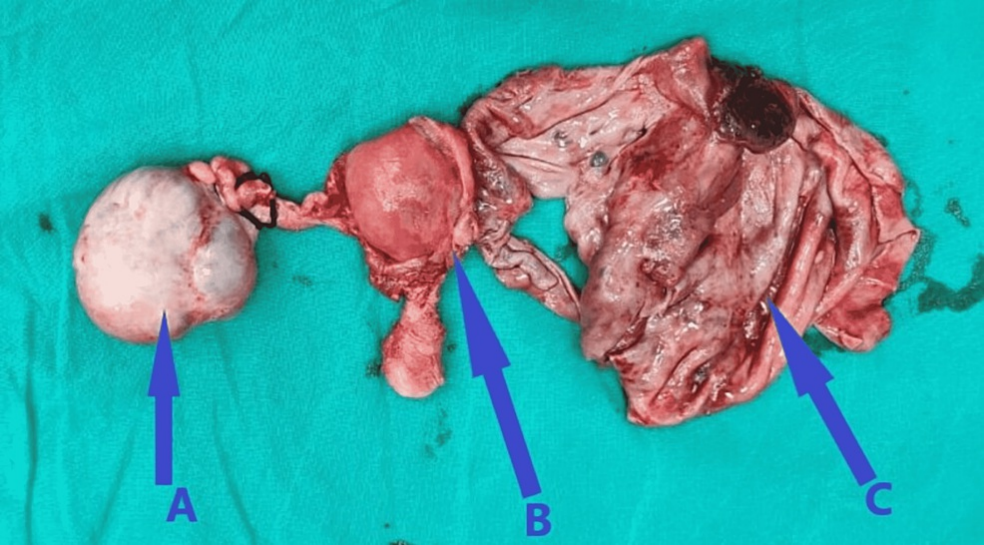
Resected surgical specimen of bilateral ovarian tumor: (A) right ovarian cystadenocarcinoma; (B) uterus; (C) left ruptured ovarian cystadenocarcinoma.

The patient was taken for exploratory laparotomy. A vertical incision of 10 cm was made and the abdomen was opened in layers so as to expose the maximum area. In order to assess the extent of the primary tumor and metastatic disease, the peritoneal cavity and retroperitoneum were thoroughly inspected and palpated. All abdominal viscera were palpated and checked, and adhesions between sigmoid and left corn structures were lysed. Ureters were identified and placed away. Dissection of mass, uterus, cervix, bilateral fallopian tube, and ovaries was done by retroperitoneal approach and sent for frozen section according to which it was labeled as bilateral papillary cystadenocarcinoma. Then retroperitoneal resection was done. Bilateral pelvic lymph nodes were removed and an omentectomy was done. No para-aortic lymph nodes were identified. The bowel was inspected for injury. Drain was inserted and hemostasis was achieved. Rectus was closed with prolene 1-0. All laparotomy sponges and instruments were removed from the abdomen and counted. The abdomen was closed in layers with Vicryl 1. Skin closure was done in layers with Ethilon 2-0 mattress sutures. The patient withstood the procedure satisfactorily and was shifted to the postoperative ward. Specimens were sent for histopathology, which confirmed bilateral serous cystadenocarcinoma. The specimen is shown in Figure 3.

The patient was discharged on postoperative day 12 after suture removal and advised to follow up every month for the first three months and then every three months for the next one year. On her last follow-up, the patient was better clinically and had no signs of recurrence.

## Discussion

**Figure 4:**
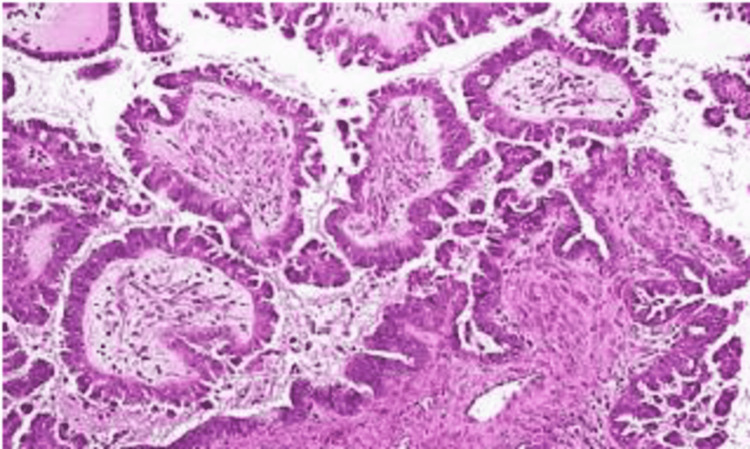
Histopathological appearance of serous cystadenocarcinoma of the ovary.

Serous papillary cystadenocarcinoma [5] is one of the most difficult cases in surgical oncology. It is one of the most common ovarian tumors and usually appears at a later stage due to its aggressive nature, the time it takes to identify, and its presentation [1,2]. Serous cystadenocarcinoma appears as a multilocular cystic ovarian neoplasm with papillary projections under the microscope (Figure 4). Psammomatous bodies, which are indicative of papillary cystadenocarcinoma of the ovary, are identified in 30% of cases on histology. The malignancy is bilateral in 25% of cases of ovarian cancer [3].

CA-125 remains the only tumor marker that is indicated for use as a diagnostic or prognostic prediction, as well as for monitoring chemotherapy efficacy and cancer recurrence after surgery [9]. CA-125 is widely used to evaluate chemotherapy response and is usually used to determine the resectability of interval debulking surgery (IDS) in patients with advanced ovarian cancer, along with cross-sectional radiological findings. CA-125 reduction may be used to predict the chemotherapy response in the neoadjuvant environment. Patients who do not respond to CA-125 also do not experience IDS and have a poor prognosis [1-6].

Ovarian cancer is commonly diagnosed at very advanced stages. Improved survival is achieved through complete debulking surgery and chemotherapy. Conventionally, NACT was introduced for unresectable tumors to reduce tumor load and perform a unique complete surgery. Many randomized control trials have been done to compare primary debulking with neoadjuvant chemotherapy. According to some studies, similar survival is seen following NACT and interval debulking surgery or primary debulking surgery. Surgical morbidity is reduced after interval debulking as compared to primary debulking surgery. Conventional drugs used are carboplatin and paclitaxel. The clinical safety of bevacizumab was also evaluated in phase 2 trials associated with some conventional drugs [10].

For women over 65 years of age, the increased prevalence of this cancer is more pronounced. The median diagnostic age is 50 to 79 years, according to previous studies [1]. A precursor to malignant ovarian tumors can be some forms of benign ovarian cysts. In addition, complex ovarian cysts significantly increase the risk of malignancy post-menopause.

A family history of breast or ovarian cancer is the most important risk factor for ovarian cancer. An elevated risk of ovarian cancer is linked to a personal history of breast cancer. Mutations in the tumor suppressor gene 108 cause more than a fifth of ovarian malignancies, whereas germline mutations in the BRCA gene 109 cause 65-85% of inherited ovarian tumors [8,9]. By the age of 40, carriers of the BRCA1 and BRCA2 mutations have a risk of ovarian cancer of less than 3%, but by the age of 50, the risk increases to 10%. Lynch syndrome accounts for 10-15% of the total cases of hereditary ovarian cancer, and in individuals with a family history of Lynch syndrome, the lifetime incidence of this cancer is 6-8% [8,9].

The advancement of neoadjuvant therapy for ovarian cancer treatment may have several purposes. Firstly, the main goal of NACT is to decrease the tumor load, thereby allowing a surgeon less extensive surgical intervention. Secondly, neoadjuvant therapy is considered to be an in-vivo test for the identification of tumor sensitivity for a given drug protocol, which further helps to manipulate treatment options. Also, neoadjuvant therapy can become essential for the trials of novel anticancer agents as it has been dealing with chemo-naive malignant states and provides an opportunity to evaluate operatively excised drug-exposed tumor tissue [11].

Overall, there is not much variation with respect to management protocols: use of the double agents consisting of carboplatin and paclitaxel is currently the only widely accepted protocol. NACT is considered the treatment of choice primarily for those patients, who cannot be subjected to complete surgical debulking, which may be due to large-scale disease spread, or because of the high risk of critical peri-operative complications [12]. The response to primary NACT largely depends on ovarian cancer histological type [13]. The current dilemma in clinical practice is whether to go for primary surgery or use NACT previous to debulking surgery. Primary debulking surgery, particularly when little to no residual disease is left behind, has frequently been recognized as one of the critical elements in improving survival in patients with advanced ovarian cancer. In addition to better patient-reported outcomes and quality-of-life (QOL) measurements, some practitioners who advocate a primary NACT followed by surgery approach have noted an increased possibility of leaving no macroscopic illness after surgery [12].

With the help of six cycles of NACT, the stage was downgraded from 4A to 2B. Then bilateral oophorectomy, total hysterectomy with lymph node dissection, and omental resection were done. Our patient was able to overcome her cancer thanks to this approach, and she was able to return home in a relatively healthy state. 

## Conclusions

The patient survival rate is improved with the NACT technique accompanied by IDS and results in successful resolution of malignant lesions. Key points to learn from this particular case were first, NACT is an effective technique in the management of advanced epithelial ovarian cancers as it not only reduces the tumor load but also helps in the downgrading of cancer stage. IDS should be carried out after primary NACT. It significantly reduces the disease related morbidity and mortality. Patient selection should be done on case to case basis. With the advancements in NACT technique, a specific protocol should be made for each patient on case to case basis.
